# The Relative Understanding of Stigma About Health (RUSH) Study: The Role of Controllability and Knowledge in Explaining Condition-Specific Variability

**DOI:** 10.1007/s10597-025-01544-y

**Published:** 2025-10-28

**Authors:** Charlotte S. Zell, Steven R. Thorp, Kenneth J. Thompson

**Affiliations:** 1 University of Houston, Houston, TX USA; 2 Alliant International University, San Diego, CA USA

**Keywords:** Stereotypes, Prejudice, Discrimination, Mental health, Physical health

## Abstract

Members of the U.S. public report negative stereotypes, prejudice, and discrimination towards individuals with a variety of health issues. The present study aimed to identify which health conditions and behaviors are most stigmatized and shed light on the factors that drive variation in stigma. A large, nationally representative sample of U.S. adults (*N* = 1,096) provided valid online survey responses to the Relative Understanding of Stigma about Health Index (RUSH-I), a novel instrument that assesses perceptions of controllability, knowledge, and perceptions of public stigma for 36 different conditions and behaviors. We hypothesized a positive relationship between controllability and stigma and an inverse relationship between knowledge and stigma. As hypothesized, stigma was substantially higher among conditions and behaviors that participants perceived as more controllable and about which they reported less knowledge. Controllability and knowledge accounted for over two-thirds of the variance in stigma across health issues. At the individual level, however, knowledge was *positively* related to stigma: Participants who reported more knowledge about a health condition or behavior tended to perceive greater stigma associated with it. Results showed that mental health conditions were generally more stigmatized than physical health conditions. Obesity was a notable exception, ranking as the most stigmatized health condition overall. These findings may help guide public health messaging, especially which conditions should be targets for stigma intervention. The findings also highlight important areas for further research, including elaborating the relationship between knowledge and stigma at both the individual and condition level.

“Stigma” initially referred to a literal mark cut or burnt into the skin of members of devalued groups to confer social judgement (Hinshaw, 2007). In modern society, stigma has become any “attribute that is deeply discrediting,” reducing its bearer from “a whole.. person to a tainted, discounted one” (Goffman, 1963). Reactions to those with such an attribute, known as public stigma, has cognitive, affective, and behavioral components.

Surveys reveal negative cognitive stereotypes, affective responses, and active discrimination toward individuals with a range of physical and mental health conditions (Pescosolido et al., 2021; Van Brakel, 2006). Research has consistently demonstrated that such reactions harm individuals with stigmatized conditions. Stigma is associated with lower rates of help-seeking, poorer engagement in treatment, and worse treatment outcomes (Akin-Odanye & Husman, 2021; Clement et al., 2015; Corrigan et al., 2014; Crapanzano et al., 2018).

A prominent theme in existing comparative stigma research is that, though both physical and mental disorders are commonly stigmatized, the public views mental health conditions more negatively (Britt, 2000; Socall & Holtgraves, 1992; Weiner et al., 1988). The cause of impairment for those with a mental illness is typically invisible and somewhat abstract. Though such relative invisibility may make it easier for individuals to hide their condition and thus avoid stigmatization, it also results in their symptoms being taken less seriously and eliciting less sympathy from the public (Corrigan et al., 2000). Variation in stigma may also be due to perceptions of controllability and knowledge about disorders.

Experimental work has demonstrated a causal link between perceptions of controllability and stigma; interventions aimed at changing perceptions about the controllability of disorders and behaviors can change affective responses to and behavioral judgments about them (Weiner et al., 1988). Research has also found that the public tends to view some disorders as more controllable than others. Among physical health conditions, HIV and obesity are seen as much more controllable than, for example, cancer and blindness (Hilbert et al., 2008; Weiner et al., 1988). Among mental health conditions, eating disorders and substance use disorders are viewed as more controllable than schizophrenia or depression (Angermeyer & Matschinger, 2004; Crisp et al., 2000). On average, mental health conditions are viewed as more controllable than physical health conditions, though there are some exceptions—for example, AIDS is viewed as more controllable than depression (Corrigan et al., 2000).

Previous research has demonstrated that a lack of familiarity and accurate knowledge about health conditions also plays a role in perpetuating stigma. Knowledge about mental health conditions may come from a variety of sources, including exposure to educational content, contact with people with mental health conditions, and personal experiences with mental health symptoms. The contact hypothesis (Allport, 1954) posits that interpersonal interactions with members of stigmatized groups can reduce prejudice by challenging inaccurate stereotypes. A substantial body of research supports this in the context of mental health stigma, with systematic reviews and meta-analyses demonstrating that interventions involving video and in particular face-to-face contact are effective in reducing stigma (Corrigan et al., 2012; Thornicroft et al., 2016; Griffiths et al., 2014). Purely educational interventions (i.e., providing accurate information about a condition but not contact with someone who holds it) have also been shown to decrease stigma. For instance, a review of 72 studies found that contact and education interventions yield modest effects on attitudes and behavioral intentions, with contact outperforming education in adults and education outperforming contact in adolescents (Corrigan et al., 2012). While not directly manipulable through intervention, personal experience with mental illness (whether one’s own or through close contacts) has also been shown to be inversely associated with stigma (Corrigan et al., 2001). Education and direct contact or personal experience may also complement each other, with knowledge providing a cognitive framework through which to understand interactions/experiences, and are often combined in stigma interventions to best increase understanding of health conditions (Henderson et al., 2014; Rüsch et al., 2005).

Studies comparing knowledge of mental health conditions have revealed dramatic variation. In a study that presented participants with vignettes describing individuals with different (unlabeled) psychiatric disorders, though 73% accurately identified depression and 66% correctly labeled schizophrenia, only 2% correctly identified borderline personality disorder (BPD; Furnham et al., 2015). In another study that presented vignettes of nine mental disorders to British participants, accurate identification rates ranged from 75% for obsessive-compulsive disorder (OCD) to 2% for social anxiety disorder (Loo et al., 2012).

Thus, both perceived controllability and knowledge appear to be causally related to health stigma, and both of these factors vary widely by diagnosis. Still, there is little empirical research regarding the implications of this variation for which conditions are least and most stigmatized. Weiner et al. (1988) compared 10 (mostly physical) conditions and behaviors and found that those perceived as less controllable elicited significantly more positive affective and behavioral responses. Specifically, cancer and heart disease (viewed as less controllable) provoked the most positive affective responses, and drug abuse and child abuse (viewed as more controllable) the least positive. Respondents similarly reported the most desire to help with cancer and blindness and the least desire to help with perpetration of child abuse and obesity.

A more recent study (Hazell et al., 2022) examined the relative stigmatization of psychiatric diagnoses and the relationship between perceptions of personal responsibility and stigma at the individual level (but not at the group/diagnosis level). In other words, the authors investigated whether a respondent’s perception of the degree to which sufferers are responsible for having a given condition was related to their endorsement of stigma about that condition. Among nine anxiety, mood, personality, dissociative, and psychotic disorders, respondents in the study desired the least social distance (the study’s operationalization of stigma) from individuals with depression, generalized anxiety disorder, and OCD; respondents desired the most social distance from those with BPD, antisocial personality disorder (ASPD), and schizophrenia. These researchers found that perceptions of personal responsibility were significantly related to stigma for posttraumatic stress disorder (PTSD) and schizophrenia but not for the other diagnoses. They did not investigate whether average differences in perceived personal responsibility among diagnoses predicted average differences in stigma among those diagnoses.

One study found that, among depression, panic attacks, eating disorders, “alcoholism,” “drug addiction,” schizophrenia, and dementia, participants had the most negative views of people with alcoholism, drug addiction, and schizophrenia (Crisp et al., 2000), though the role of condition-specific controllability and knowledge was not investigated. Additional research confirms that the public holds more negative stereotypes about psychotic disorders and substance use disorders (SUDs) than mood or anxiety disorders (Barry et al., 2014; Corrigan et al., 2009; Pescosolido et al., 1999; Wood et al., 2014). In summary, findings on the relative stigmatization of psychiatric disorders appear to correspond to findings on public knowledge and perceptions of controllability, with the notable exception of schizophrenia. However, the value of these factors in explaining variation in diagnosis-specific stigma remains untested. In addition, existing survey research has only compared stigma among a relatively small number of health issues. The only existing work to our knowledge that has compared stigma among a large number of disorders was a computational analysis of news articles (Best & Arseniev-Koehler, 2023). This study examined media stigma across over 100 health issues, the large majority of which were chronic physical health conditions. While their finding that behavioral health conditions were more stigmatized than physical health conditions is consistent with survey research, their findings regarding the relative stigmatization of various behavioral health conditions departed substantially from those of prior survey-based studies. For instance, they found that depression was the most stigmatized among all behavioral health conditions, ranking above even drug addiction and anorexia. These discrepancies suggest that, while important, stigma in media may not consistently reflect the views of the general public and be shaped by somewhat different factors.

Against this backdrop, the goal of the Relative Understanding of Stigma about Health (RUSH) study is to shed light on public stigma (a “rush to judgment”), which can, in turn, guide more targeted and effective interventions. The present paper is focused on identifying which health conditions are perceived by the public as least and most stigmatized and on understanding some of the factors that may drive this variation. We investigated relative perceived stigma among 36 conditions and behaviors (see Table 1) in a large sample. These conditions include cancer, diabetes, obesity, headaches, Alzheimer’s disease, depression, eating disorders, SUDs, social anxiety disorder, PTSD, BPD, schizophrenia, and pedophilia, as well as perpetrating and experiencing acts such as intimate partner violence (IPV), child abuse, and sexual assault. The RUSH study is the first to examine the role of both knowledge and perceived controllability in predicting the relative stigmatization of a wide range of physical and mental health issues. We also investigated whether these factors are associated with perceived stigma at the individual level for each of the 36 conditions and behaviors.

Based on prior cross-sectional and experimental research demonstrating the link between controllability and stigma, we hypothesized that perceived stigma would generally be higher among conditions and behaviors that are perceived as more controllable. Though knowledge has not been previously applied to explaining the *relative* stigmatization of health issues, the inverse relationship between knowledge and stigma (that is, more knowledge is associated with less stigma) has been well-established in the stigma intervention literature. Therefore, we hypothesized that perceived stigma would be lower among conditions and behaviors about which participants report more knowledge. We expected to find the same relationships at the individual level—that is, individuals’ perceptions of the controllability of a given health condition or behavior would be positively related to their perceptions of public stigma about it, while their reported knowledge about a given health condition or behavior would be negatively related to their perceptions of public stigma about it. Though we anticipated overlap in the stigma rankings of physical and mental health conditions, we predicted that, on average, mental health conditions would be more stigmatized than physical health conditions. Finally, we expected that perpetrating a harmful behavior would be perceived as more stigmatized than experiencing or being a survivor of that behavior.

## Methods

### Measures

The Relative Understanding of Stigma about Health Index (RUSH-I) comprises three 36-item self-report scales that assess (a) knowledge, (b) perceptions of controllability, and (c) perceptions of public stigma about a variety of health conditions and behaviors. Items are measured on a five-point Likert scale (0–4). Many existing measures provide more comprehensive evaluations of each of these constructs for specific health conditions or mental illness in general but are too lengthy and time-consuming to administer for dozens of different conditions without substantial burden on respondents (e.g., asking respondents to answer questions about a detailed vignette for each of 36 conditions). The RUSH-I was developed to provide a quick and straightforward assessment of large number of health conditions and behaviors. The measures in the RUSH-I were designed from the authors’ clinical experience to represent the range of stigma about healthcare, but the conditions which were included were not empirically derived. The knowledge scale of the RUSH-I asks:

How much knowledge do you have about each of the following conditions or behaviors, in terms of people you know, facts you’ve learned, or your personal experience? For “experiencing” events, how much do you know about people who have experienced those events? For “perpetration” events, how much do you know about people who have committed the act?

Response options range from *no knowledge* to *a great deal of knowledge*. While personal contact or experiences with health conditions and exposure to factual knowledge about them are separable, we chose to combine them into a single scale based on the recognition that knowledge of conditions may be obtained from a variety of sources, and previous literature showing that factual and experiential sources of knowledge are interrelated and have similar implications for stigma (Corrigan et al., 2012; Rüsch et al., 2005).

The controllability scale of the RUSH-I asks:

How much do you think each of the following conditions or behaviors can be controlled by the person who has it or does it? For “experiencing” events, how much can the person control what happened to them? For “perpetration” events, how much can the person who committed the act control it?

Response options range from *they have no control* to *they can control completely.*

The stigma scale of the RUSH-I asks:

How much stigma is in our society about the following conditions or behaviors? Stigma means negative stereotypes, prejudice, and discriminatory behavior.

Response options range from *no stigma* to *a great deal of stigma*. Although stigma is often assessed via measures of endorsed stereotypes or desire for social distance (Link et al., 1987; Martin et al., 2000), perceptions of public stigma are also widely studied and predict important outcomes, including self-stigma and willingness to seek help (Rosenfield, 1997; Griffiths et al., 2008; Skopp et al., 2012; Vogel et al., 2013). We chose this approach because it provides a parsimonious and scalable assessment across dozens of conditions, while capturing societal attitudes that are central to understanding stigma as a cultural phenomenon.

In addition to the RUSH-I, participants completed a 14-item version of the Physical Health Questionnaire (Schat et al. 2005); a modified version of the Military Stigma Scale (MSS; Skopp et al., 2012); the Depression, Anxiety, Stress Scale (DASS-21; Loviband & Loviband, 1995); and a modified version of the Life Events Checklist for *DSM-5* (LEC-5; Weathers et al., 2013a, 2013b), in combination with the PTSD Checklist for *DSM-5* (PCL-5 Weathers et al., 2013a, 2013b). However, data regarding those measures will be reported elsewhere.

### Procedure

This study was approved by the [masked institution] Institutional Review Board (Protocol #124258). Informed consent was obtained from all participants prior to their involvement in the study, and ethical research procedures were followed. A nationally representative sample of U.S. adults was recruited for the RUSH study using Qualtrics panels. The study hypotheses and analysis plan were pre-registered with the Open Science Framework (OSF) on October 18, 2023, to promote transparency: https://osf.io/6rg8y/?view_only=61e0755a12af450db35c7354c62b59f3.

Inclusion criteria were U.S. residency, self-reported English fluency, and access to the internet. Qualtrics recorded 1994 initial survey responses.

**Fig. 1:**
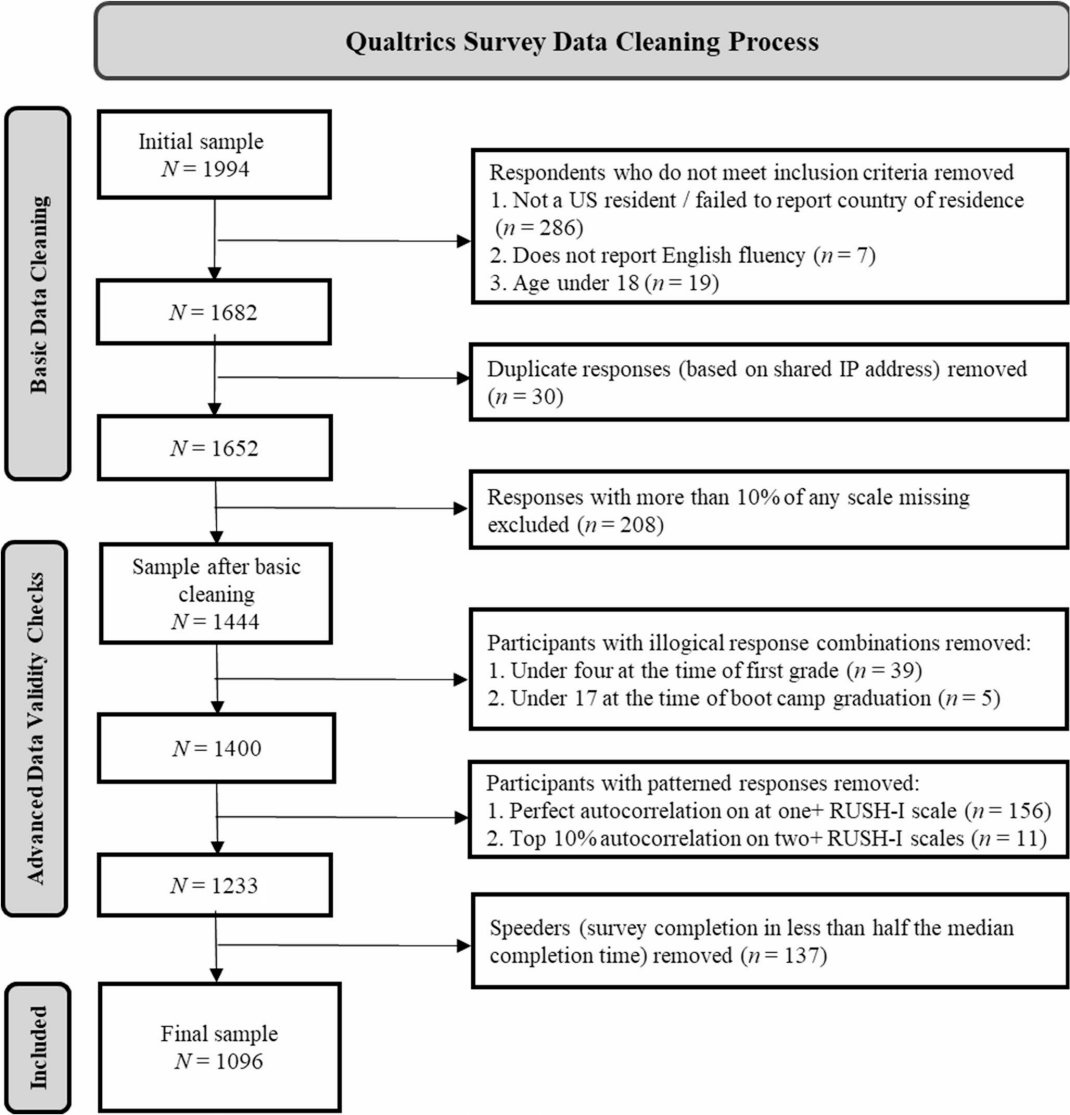
Data Cleaning Process. *Note.* Many participants failed multiple checks; for instance, a participant might not report being a US resident, report being under 18, and be a speeder. However, they would only be counted towards the number of participants excluded for not being a US resident (the first of the three checks that they failed)

Figure 1 depicts the data cleaning process. In a first round of data cleaning, we confirmed that participants met inclusion criteria and checked for duplicates and missing data. Demographic questions assessed whether participants met inclusion criteria. Respondents who did not report U.S. residency, English fluency, or meeting the minimum age of 18 were removed from the sample. For duplicate entries, we retained the first complete entry (unless different responses to demographic questions were provided or both entries were completed on the same day, suggestive of deceptive/unreliable responding). Participants who did not finish the survey or failed to respond to more than 10% of items on any scale were excluded. This first round of data cleaning reduced the sample size from 1,994 to 1,444.

A second round of data cleaning involved more sophisticated data validity checks aimed at detecting inattentive responding. Prior research has found that, when asked to randomly generate a sequence of numbers, participants tend to provide highly patterned responses (Baddeley et al., 1998). Inattentive survey respondents also often flatline measures by providing the same response repeatedly (Johnson, 2005). Attentive participants, on the other hand, are unlikely to generate responses that follow repetitive patterns. We identified participants with repetitive response patterns using the autocorrelation screening tool developed by Gottfried et al. (2022). To capture patterns, the tool calculates correlations between each participant’s responses and copies of their responses that are shifted/lagged. On each RUSH-I scale, approximately 5–7% of participants had perfectly autocorrelated responses, mostly due to flatlining. These participants were removed, as were participants whose autocorrelations were in the top 10% of the sample on at least two RUSH-I scales.

Remaining participants were excluded for inconsistent responses to questions pertaining to age and years of education (e.g., reported years of education exceeding reported age). For a veteran subgroup of the sample, we similarly excluded participants whose responses to age and reported years of veteran status were inconsistent with a calculated age of 17 (the minimum, waiver-required age for service entrance) at the time of boot camp graduation. Finally, we removed participants for “speeding,” which we operationalized as completing the survey in less than half of the median completion time. This second round of data cleaning reduced the number of participants from 1,444 to a final sample of 1,096. Missing values on the RUSH-I (10 out of 118,368 total values) in the final sample were replaced using mean imputation. Some minor demographic differences were observed between excluded and retained participants. Compared to those excluded, retained respondents were an average of around four years older (*p >*.001), had a higher household income (*p* =.022), and were more likely to identify as White (*p* =.017). Excluded and retained respondents did not significantly differ in terms of gender, ethnicity, years of education, or military status.

### Statistical Analysis

The relationship between controllability and perceived stigma, as well as knowledge and perceived stigma, was assessed at both the group/condition level and the individual level, using linear regression analysis. Group level analysis aimed to identify whether average differences in knowledge about conditions and perceptions of their controllability explained average differences in stigma among conditions. For group level analysis only, RUSH-I response data were restructured into three new variables (one variable for each RUSH-I scale) with 36 rows (one row for each condition/behavior). Each datapoint on the stigma scale, for instance, contained the mean stigma rating across all 1,096 participants for a specific condition or behavior. Two simple linear regressions were performed using these restructured data to test whether (a) perceived controllability by condition/behavior or (b) knowledge by condition/behavior predicted stigma by condition/behavior. A multiple regression with both controllability and knowledge as predictors was conducted to determine total variance in stigma among conditions/behaviors that these two variables can explain.

Individual level analysis sought to determine whether an individual’s perception of the controllability of a given condition and reported knowledge of it were related to the perception of stigma about that condition. Especially at the individual level, it is plausible that knowledge and perception of controllability might be causally related. For instance, respondents’ knowledge about a condition may affect their beliefs about its controllability. Given that knowledge and stigma are likely to be causally related to each other, the relationship between controllability and stigma was assessed separately from the relationship between knowledge and stigma. This decision was made a priori based on recommendations in the statistical literature that endogeneity bias arises when predictors are causally related, even in the absence of problematic multicollinearity indices (Wooldridge, 2010). We conducted 39 simple linear regressions examining the relationship between the perception of controllability and stigma, one regression for each condition/behavior and three additional regressions analyzing the relationship between a participant’s (a) average perception of controllability and stigma generally, (b) average perception of controllability and stigma about mental health, and (c) average perception of controllability and stigma about physical health. An additional 39 simple linear regressions similarly probed the relationship between knowledge and stigma. Average controllability, knowledge, and stigma of index subcomponents (e.g., mental health) were operationalized as the mean response to all items on the respective scales of the RUSH-I for the applicable scale components. For instance, average mental health knowledge was operationalized as the mean response to all items about mental health conditions on the knowledge scale of the RUSH-I.

To determine whether mental illness is more stigmatized than physical illness, we calculated each respondent’s average stigma rating across all mental health conditions (alcohol use disorder [AUD], ASPD, ADHD, bipolar disorder, BPD, depression, eating disorders, neurodevelopmental disorders including autism and intellectual disability, OCD, panic disorder, specific phobias, PTSD, schizophrenia and psychotic disorders, social anxiety disorder, SUD with illicit substances, SUD with prescribed substances, pedophilia fantasy without perpetration, and sexual assault fantasy without perpetration) and average stigma rating across all physical health conditions (cancer, diabetes, headaches, and obesity or being overweight). The means of these new variables across all participants were compared using a paired samples *t*-test. Paired samples *t*-tests were also used to compare perceived stigma about perpetrating a behavior to perceived stigma about being the victim/survivor of that behavior. Separate *t*-tests were conducted for each type of behavior (IPV, child neglect, child physical abuse, child sexual abuse, and sexual assault or rape).

A series of one-way ANOVAs and paired samples *t*-tests were conducted to explore the relative stigmatization of various conditions and behaviors. To reduce the number of factors per ANOVA, items were separated into five categories: physical health, mental health, “mixed” conditions with both physical and mental health components (Alzheimer’s disease, insomnia, and older adulthood issues), perpetration, and experiencing. Tobacco use was included along with SUDs in the mental health category. This decision was made for consistency across substance-related items and is consistent with addiction literature that conceptualizes tobacco use as part of the continuum of addictive behaviors (West & Brown, 2013). Stigma studies have similarly examined tobacco use alongside alcohol and illicit substances when examining public attitudes towards addictive behaviors and conditions (Olsen et al., 2003; Room, 2005). A first ANOVA compared mean stigma across the five categories. Additional ANOVAs compared stigma among items within each category. Mental health conditions were further subdivided into 10 groups: anxiety disorders and OCD (four items), substance use (four items), paraphilias (two items), personality disorders (two items), neurodevelopmental disorders (two items), schizophrenia/psychotic disorders (one item), unipolar depression (one item), bipolar disorder (one item), eating disorders (one item), and PTSD (one item). An ANOVA examined differences in stigma across the 10 subcategories of mental health conditions. Additional ANOVAs and *t*-tests compared stigma within subcategories.

All hypothesis tests were two-tailed with a critical alpha of 0.05. We used the Benjamini-Hochberg procedure for False Discovery Rate (FDR) correction to account for multiple hypothesis tests. Global FDR correction was performed based on the total of 187 tests conducted (including all pairwise comparisons—the ANOVA with 10 levels, for instance, constituted 45 pairwise comparisons).

## Results

### Sample Characteristics

Participants (*N* = 1,096) were 52.1% male, 47.8% female, and 0.1% another gender. The sample was 73.1% White, 14.5% Black, 5.7% Asian American, 1.9% American Indian or Native American, 0.5% Native Hawaiian or Other Pacific Islander, and 1.9% Biracial or Multiracial. Additionally, 2.2% of respondents indicated that they were another race or preferred not to disclose their race. Nearly one-fifth of the sample (18.1%) identified as Hispanic or Latinx, while another 2.0% stated that they preferred not to disclose their ethnicity. The sample had a mean age of 44.5 years (*SD* = 17.8), with 15.2 years (*SD* = 5.2) of formal education. The median household income range of the sample was $60,000 to $79,999. Current or former military service was reported by 16.9% of respondents. Overall, the final sample was demographically diverse and approximately representative of the U.S. adult population.

### Do Perceived Controllability and Knowledge Predict Condition-Specific Stigma?

**Fig. 2:**
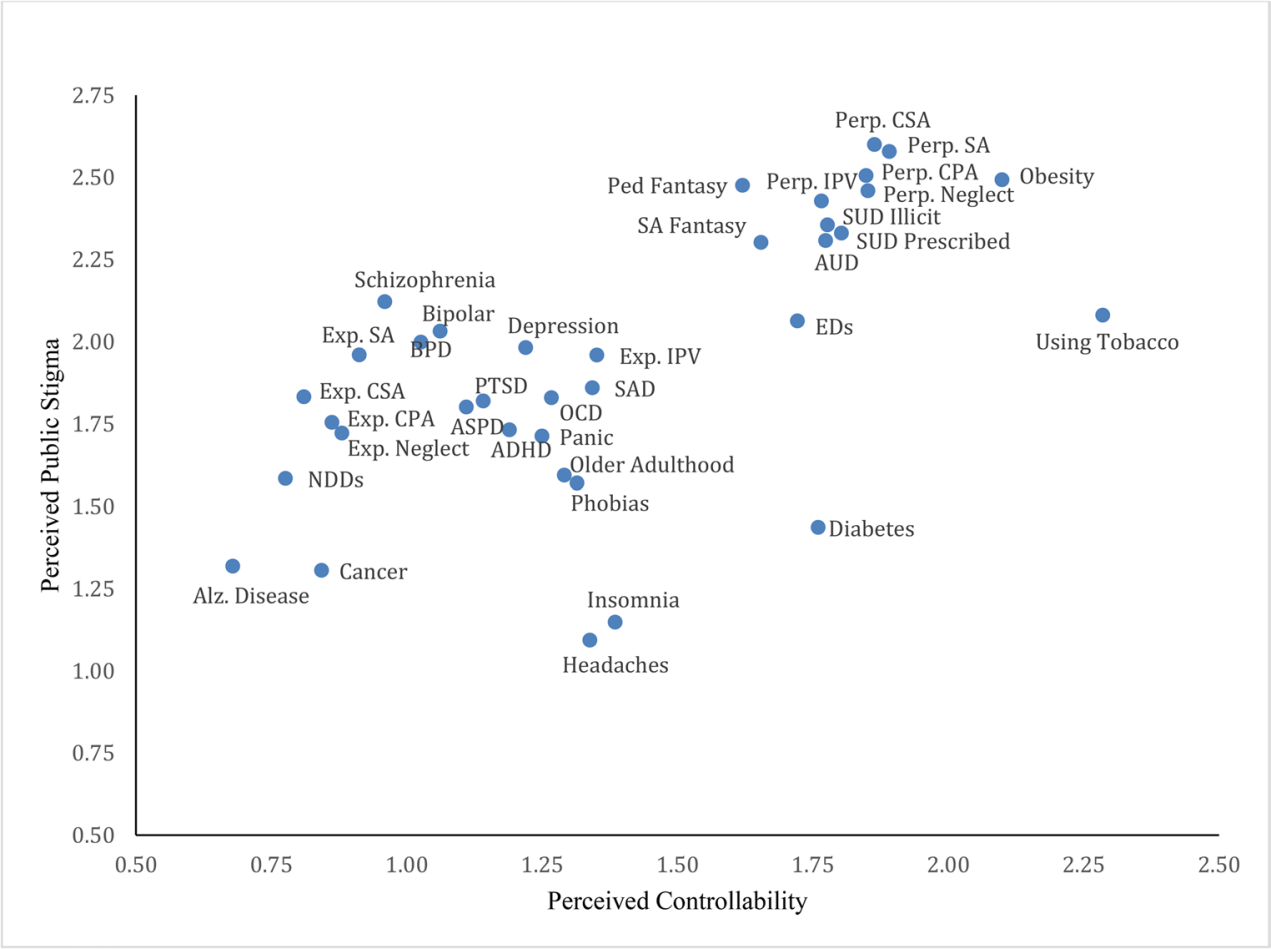
Relationship Between Perceived Controllability and Perceived Public Stigma at the Condition Level. *Note. *AUD = Alcohol Use Disorder; Alz. Disease = Alzheimer’s Disease; ASPD = Antisocial Personality Disorder; BPD = Borderline Personality Disorder; CPA = Child Physical Abuse; CSA = Child Sexual Abuse EDs = Eating Disorders; Exp. = Experiencing; IPV = Intimate Partner Violence; NDDs = Neurodevelopmental Disorders; OCD= Obsessive-Compulsive Disorder; Perp. = Perpetration; PTSD = Posttraumatic Stress Disorder; SA = Sexual Assault; SAD = Social Anxiety Disorder; SUD = Substance Use Disorder; ADHD = attention-deficit/hyperactivity disorder

**Fig. 3:**
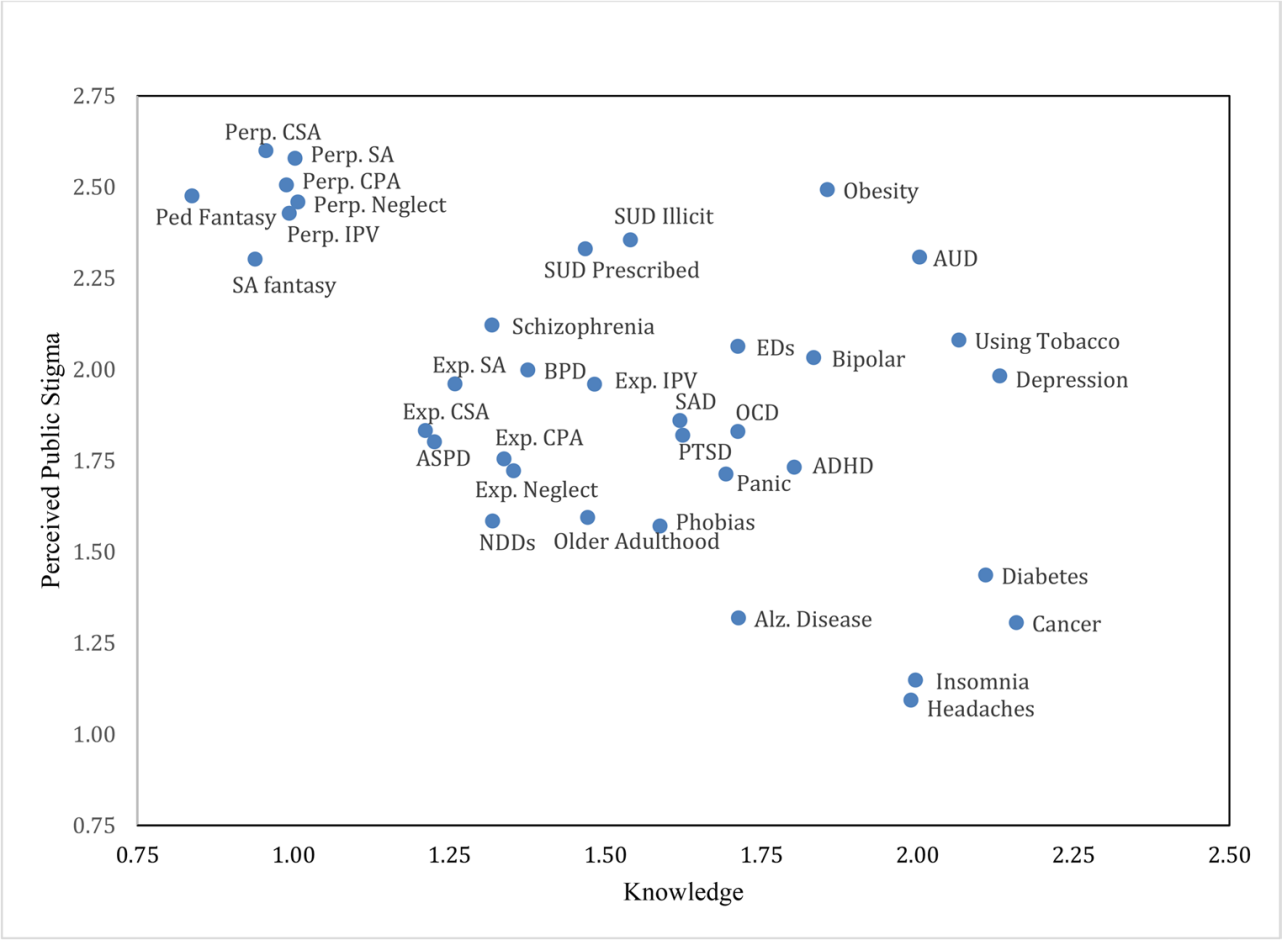
Relationship Between Knowledge and Perceived Public Stigma at the Condition Level. *Note. *AUD = Alcohol Use Disorder; Alz. Disease = Alzheimer’s Disease; ASPD = Antisocial Personality Disorder; BPD = Borderline Personality Disorder; CPA = Child Physical Abuse; CSA = Child Sexual Abuse EDs = Eating Disorders; Exp. = Experiencing; IPV = Intimate Partner Violence; NDDs = Neurodevelopmental Disorders; OCD = Obsessive-Compulsive Disorder; Perp. = Perpetration; PTSD = Posttraumatic Stress Disorder; SA = Sexual Assault; SAD = Social Anxiety Disorder; SUD = Substance Use Disorder; ADHD = attention-deficit/hyperactivity disorder

Results of the regression analysis indicated that differences in the perceived controllability and knowledge of health conditions/behaviors strongly predicted differences in stigma. As hypothesized, stigma was significantly higher among conditions and behaviors that participants perceived as more controllable (*r* =.62, *p* <.001). Perceived controllability accounted for 38.9% of the variance in stigma among conditions and behaviors. Figure 2 displays the relationship between controllability and stigma at the condition level. Notably, *all* highly stigmatized conditions/behaviors also ranked high in controllability. As expected, perceived stigma was significantly lower among conditions and behaviors about which participants reported greater knowledge (*r* = −.57, *p* <.001). Knowledge alone explained 32.7% of the variance in stigma among RUSH-I items. Figure 3 displays the relationship between knowledge and stigma at the condition level. As shown in Fig. 3, *all* conditions/behaviors that ranked low in knowledge ranked high in stigma.

In the multiple regression, both controllability and knowledge remained strong independent predictors of stigma (β_controllability_ = 0.59, *p* <.001; β_knowledge_ = − 0.53 *p* <.001). The coefficients of the predictors changed only slightly from the bivariate to the multivariate regression because knowledge by condition/behavior was not significantly related to perceived controllability by condition/behavior (*r* =.06, *p =*.71). Together, controllability and knowledge explained 67.3% of the variance in stigma among RUSH-I items. Examination of predicted and observed values showed that the multiple regression closely predicted stigma about most conditions and behaviors. However, stigma about headaches, diabetes, and insomnia was substantially lower than expected, while stigma about depression, bipolar disorder, and AUD was notably higher than predicted. On average, the regression model overestimated stigma about physical health conditions (mean residual = − 0.15) and slightly underestimated stigma about mental health conditions (mean residual = 0.08).

### Is Perception of Controllability or Knowledge Related To Stigma at the Individual Level?

Individual-level perceptions of controllability and knowledge were significantly associated with perceptions of stigma. Table 1 reports the correlation coefficients for controllability versus stigma and knowledge versus stigma for each condition/behavior. Perceived controllability was significantly positively related to stigma at the individual level for all conditions and behaviors. Respondents’ perceptions of the controllability of Alzheimer’s disease, perpetration of neglect of a child, and perpetration of physical abuse of a child were particularly strongly associated with their perceptions of stigma about these issues. The relationships between average *overall* controllability and average stigma (*r* =.409, *p* <.001), average *mental health* controllability and average mental health stigma (*r* =.335, *p* <.001), and average *physical health* controllability and average physical health stigma (*r* =.309, *p* <.001) were also highly significant. Overall, individual- and group-level findings about the relationship between controllability and stigma were consistent, though the relationship was stronger at the group level.

Contrary to our prediction, individual knowledge was positively associated with stigma for all items except the five perpetration behaviors and pedophilia fantasy without perpetration. Reported knowledge was especially strongly predictive of perceived stigma for panic disorder and older adulthood difficulties. There were also significant relationships between average *overall* knowledge and average stigma (*r* =.444, *p* <.001), average *mental health* knowledge and average mental health stigma (*r* =.440, *p* <.001), and average *physical health* knowledge and average physical health stigma (*r* =.309, *p* <.001).

### Mental Health Stigma Versus Physical Health Stigma

As hypothesized, mental health conditions were perceived as more stigmatized than physical health conditions (*d* = 0.561, *p* <.001). The mean stigma rating among mental health conditions was 1.99 and mean stigma among physical health conditions was 1.58. Despite this average difference, the condition in the RUSH-I with the highest perceived stigma ranking was obesity. Obesity ranked higher than even pedophilia fantasy or SUDs.

### Perpetrating Versus Experiencing

For all five behaviors, perpetration was perceived as significantly more stigmatized than experiencing it as a survivor. The *p*-values for all paired samples *t*-tests were less than 0.001. Differences in stigma between perpetrating and experiencing were greatest for child sexual abuse (mean difference = 0.77, *d* = 0.47) and smallest for IPV (mean difference = 0.47, *d* = 0.32).

### Most and Least Stigmatized Conditions

All RUSH-I items are ranked from highest to lowest in perceived stigma in the first column of Table 1; the second column displays the mean stigma rating across all participants for each item. Overall, the highest average ratings of stigma were for perpetration of sexual abuse of a child, perpetration of sexual assault, perpetration of physical abuse of a child, obesity, and pedophilia fantasy without perpetration or offense. The least stigmatized items were headaches (including migraines), insomnia, cancer, Alzheimer’s disease, and diabetes.

**Table 1:** Perceived stigma, perceived controllability, and reported knowledge of health issues

Condition/Behavior: Stigma Hierarchy	Stigma Mean (SE)	Controllability Mean (SE)	Knowledge Mean (SE)	*R *(Knowledge, Stigma)	*R* (Controllability, Stigma)
1. Perpetration of Child Sexual Abuse	2.599 (0.041)	1.864 (0.044)	0.956 (0.037)	0.006	0.344**
2. Perpetration of Sexual Assault	2.578 (0.041)	1.891 (0.045)	1.003 (0.038)	0.012	0.331**
3. Perpetration of Child Physical Abuse	2.505 (0.041)	1.848 (0.044)	0.989 (0.037)	0.004	0.370**
4. Obesity or Being Overweight	2.493 (0.035)	2.099 (0.034)	1.856 (0.038)	0.237**	0.209**
5. Pedophilia Fantasy w/o Perp.	2.475 (0.040)	1.620 (0.041)	0.838 (0.035)	0.030	0.234**
6. Perpetration of Child Neglect	2.459 (0.041)	1.852 (0.044)	1.007 (0.038)	0.055	0.388**
7. Perpetration of IPV or DV	2.428 (0.040)	1.766 (0.042)	0.994 (0.037)	0.007	0.327**
8. SUD with Illicit Substances	2.355 (0.037)	1.777 (0.036)	1.540 (0.039	0.262**	0.206**
9. SUD with Prescribed Substances	2.330 (0.036)	1.803 (0.036)	1.468 (0.038)	0.215**	0.184**
10. Alcohol Use Disorder	2.307 (0.034)	1.774 (0.033)	2.004 (0.037)	0.256**	0.117**
11. Sexual Assault Fantasy w/o Perp.	2.302 (0.041)	1.654 (0.041)	0.939 (0.036)	0.077*	0.266**
12. Schizophrenia	2.121 (0.037)	0.960 (0.034)	1.318 (0.037)	0.205**	0.166**
13. Using Tobacco	2.080 (0.037)	2.286 (0.037)	2.067 (0.041)	0.208**	0.139**
14. Eating Disorders	2.063 (0.034)	1.722 (0.033)	1.713 (0.036)	0.178**	0.145**
15. Bipolar Disorder	2.032 (0.035)	1.062 (0.032)	1.834 (0.036)	0.273**	0.183**
16. Borderline Personality Disorder	1.998 (0.036)	1.026 (0.032)	1.376 (0.038)	0.273**	0.190**
17. Depression (Unipolar)	1.982 (0.035)	1.218 (0.032)	2.132 (0.039)	0.339**	0.192**
18. Experiencing Sexual Assault	1.960 (0.041)	0.912 (0.036)	1.259 (0.040)	0.226**	0.239**
19. Experiencing IPV or DV	1.959 (0.038)	1.351 (0.036)	1.483 (0.039)	0.162**	0.196**
20. Social Anxiety Disorder	1.859 (0.036)	1.343 (0.032)	1.620 (0.040)	0.302**	0.203**
21. Experiencing Child Sexual Abuse	1.832 (0.042)	0.810 (0.035)	1.212 (0.040)	0.234**	0.283**
22. Obsessive-Compulsive Disorder	1.829 (0.035)	1.267 (0.033)	1.713 (0.037)	0.245**	0.230**
23. Posttraumatic Stress Disorder	1.819 (0.035)	1.141 (0.033)	1.624 (0.039)	0.267**	0.234**
24. Antisocial Personality Disorder	1.801 (0.035)	1.110 (0.032)	1.226 (0.037)	0.314**	0.210**
25. Experiencing Child Physical Abuse	1.755 (0.041)	0.862 (0.036)	1.338 (0.040)	0.232**	0.344**
26. ADHD	1.732 (0.034)	1.190 (0.032)	1.803 (0.036)	0.283**	0.190**
27. Experiencing Child Neglect	1.722 (0.040)	0.880 (0.036)	1.353 (0.039)	0.259**	0.306**
28. Panic Disorder	1.713 (0.036)	1.250 (0.032)	1.693 (0.040)	0.353**	0.299**
29. Older Adulthood Difficulties	1.594 (0.035)	1.291 (0.033)	1.472 (0.037)	0.343**	0.287**
30. NDDs including Autism and ID	1.584 (0.037)	0.776 (0.032)	1.319 (0.036)	0.316**	0.263**
31. Specific Phobias	1.570 (0.035)	1.315 (0.032)	1.588 (0.036)	0.156**	0.224**
32. Diabetes	1.436 (0.038)	1.760 (0.036)	2.109 (0.036)	0.196**	0.155**
33. Alzheimer’s Disease	1.318 (0.037)	0.679 (0.033)	1.714 (0.033)	0.235**	0.406**
34. Cancer	1.306 (0.040)	0.843 (0.034)	2.159 (0.035)	0.135**	0.325**
35. Insomnia	1.148 (0.037)	1.385 (0.031)	1.998 (0.038)	0.222**	0.296**
36. Headaches incl. Migraines	1.093 (0.038)	1.339 (0.034)	1.990 (0.038)	0.152**	0.292**

The largest nominal *p*-value that remained less than 0.05 after FDR correction was 0.017. Therefore, 0.017 was considered the new cutoff value for statistical significance. DV = Domestic Violence; ID = Intellectual Disability; IPV = Intimate Partner Violence; NDDs = Neurodevelopmental Disorders; Perp. = Perpetration; SUD = Substance Use Disorder; ADHD = attention-deficit/hyperactivity disorder

*p <.017, **p <.001

Table 2 reports the results of the repeated measures ANOVA comparing stigma among the five broad categories of items. Condition/behavior types are ranked from most to least stigmatized, with the mean stigma across all participants and the 95% confidence interval of the mean displayed for each category. Pairwise comparisons revealed that all differences in stigma between categories were highly significant. Perpetration items ranked highest in perceived stigma, followed by mental health and then experiencing items. Mixed conditions were rated as less stigmatized than physical health conditions, a result influenced by the high stigma associated with obesity, which positively skewed the mean stigma of physical health. Excluding obesity, the mean stigma of physical health conditions (1.28) was lower than that of mixed conditions.

**Table 2:** Stigma by condition and behavior type

Condition/Behavior Type: Stigma Hierarchy	Mean Stigma	95% Confidence Interval [Lower Limit, Upper Limit]
1. Perpetration	2.514	[3.441, 3.587]
2. Mental Health	1.998	[2.950, 3.045]
3. Experiencing	1.845	[2.775, 2.915]
4. Physical Health	1.582	[2.529, 2.635]
5. Mixed Conditions	1.353	[2.295, 2.412]

Among *perpetration* items, sexual abuse of a child and sexual assault or rape received the highest stigma ratings. The difference in stigma between these two behaviors was not significant. Physical abuse of a child ranked third, followed by neglect of a child, and, finally, IPV. The difference in stigma between physical abuse of a child and neglect of a child—as well as between neglect of a child and IPV—was not significant. All other pairwise comparisons between perpetration items yielded significant results. The *survival from experiences* that were rated highest in stigma, on the other hand, were sexual assault and IPV; they did not significantly differ in stigma. Sexual abuse as a child fell in the middle in terms of perceived stigma. Physical abuse as a child and neglect as a child were perceived as the least stigmatized; the stigma gap between these two experiences was also not significant. Note that adulthood experiences were viewed as more stigmatized than analogous childhood experiences. As shown in Table 1; Fig. 2, adulthood experiences were also perceived as more controllable.

As previously mentioned, obesity was by far the most stigmatized physical health condition in the RUSH-I. Diabetes ranked second, cancer third, and headaches lowest. All differences in stigma among physical health conditions were significant. Among the three mixed conditions, perceived stigma was highest for older adulthood difficulties and lowest for insomnia. All disparities in stigma among mixed conditions were significant.

Table 3 displays the stigma hierarchy of 10 types of mental health issues. Paraphilias ranked highest in perceived stigma, followed by substance use. Mental health issues that were perceived as relatively lower in stigma were anxiety disorders and items in the neurodevelopmental disorders group (ADHD and neurodevelopmental disorders including autism and intellectual disability). All pairwise comparisons were significant except schizophrenia versus eating disorders, eating disorders versus bipolar disorder, and bipolar disorder versus unipolar depression. Examining differences in stigma within mental health subcategories, *t*-tests showed that pedophilia fantasy was significantly more stigmatized than sexual assault fantasy, BPD was significantly more stigmatized than antisocial personality disorder, and ADHD was significantly more stigmatized than neurodevelopmental disorders including autism and intellectual disability. An ANOVA of substance use items found that SUD with illicit substances, SUD with prescribed substances, and AUD did not significantly differ from one another in stigma. All three, however, were perceived as significantly more stigmatized than tobacco use. Finally, results showed that social anxiety disorder and OCD were rated significantly higher in stigma than panic disorder, which was rated significantly higher than phobias.

**Table 3:** Stigma by mental health issue type

Mental Health Stigma Hierarchy	Mean Stigma	95% Confidence Interval [Lower Limit, Upper Limit]
1. Paraphilias	2.389	[3.316, 3.461]
2. Substance Use	2.268	[3.214, 3.323]
3. Schizophrenia	2.121	[3.049, 3.194]
4. Eating Disorders	2.063	[2.996, 3.130]
5. Bipolar Disorder	2.032	[2.963, 3.101]
6. Depression	1.982	[2.912, 3.051]
7. Personality Disorders	1.900	[2.838, 2.961]
8. PTSD	1.819	[2.750, 2.889]
9. Anxiety and OCD	1.743	[2.686, 2.800]
10. Neurodevelopmental Disorders	1.658	[2.597, 2.719]

## Discussion

This study was the first to use survey data to compare public stigmas about a wide variety of health issues. We aimed to understand the role of knowledge and perceived controllability in shaping perceived public stigma, with a focus on distinguishing between findings at the condition/behavior level and the individual level of analysis. Results provide insight into the predictors of health stigma and shed light on the relative stigmatization of different conditions and behaviors. This study builds upon previous literature that has compared self-reported public perceptions and endorsements of stigma across a smaller number of health conditions and behaviors (e.g., Crisp et al., 2000; Hazell et al., 2022; Weiner et al. 1988), as well as research examining stigma in media across a large number of conditions utilizing other methods, such as computational text analysis of news articles (Best & Arseniev-Koehler, 2023).

A key finding was that perceived stigma tended to be much higher among conditions and behaviors that participants perceived as more controllable and about which they reported less knowledge. These two factors explained more than two-thirds of the variability in stigma among health issues. These results confirm the relationship between controllability and stigma identified by Weiner et al. (1988), yet with a larger sample and by comparing a greater number of health conditions and behaviors. To our knowledge, this study is the first to establish a relationship between knowledge about health issues and their relative stigmatization. Though knowledge and controllability were strong predictors of stigma, they still tended to slightly underestimate stigma among mental health conditions relative to physical health conditions. Factors such as the invisible and abstract nature of mental illness may contribute to its stigmatization.

Findings about perceived controllability at the individual level mirrored those at the group level. Surprisingly, however, individuals who reported more *knowledge* about a specific condition or behavior reported perceiving *more* stigma associated with it. This finding seemingly contradicts previous intervention studies showing that increasing people’s knowledge about mental illness reduces stigma (Corrigan et al., 2012). However, stigma intervention studies generally measure *endorsements* of stigma (e.g., negative attitudes or desired social distance), while we assessed *perceptions* of stigma in society. Individuals who possess more knowledge about a specific condition may have a greater understanding of its challenges and be more attuned to examples of societal stigmatization. This interpretation could also help explain the disparity between our findings at the individual level versus the group level. Overall, greater public discourse about a condition or behavior (reflected in greater public knowledge about it) could help reduce stigma. At the same time, more knowledgeable individuals may be more likely to notice when this discourse contains misconceptions or stigmatizing themes.

It is also important to consider that we did not objectively assess participants’ knowledge but, instead, asked them to report how much knowledge they *believed* they possessed. Relative knowledge about conditions in our sample differed somewhat from findings in previous studies that objectively measured knowledge (e.g., providing a correct diagnostic label for a collection of symptoms; Furnham et al., 2015), indicating that participants’ assessments of their own knowledge may not have been accurate. Given the prevalence of misinformation about health conditions, it is even possible that participants who had been exposed to more content about health issues (e.g., through social media) had stronger misconceptions about them. This explanation, however, does not easily account for our opposing findings at different levels of analysis. The relationship between knowledge and health stigma requires further elaboration.

Similar to previous researchers (e.g., Britt, 2000; Socall & Holtgraves, 1992; Weiner et al., 1988), we found that mental health conditions were significantly more stigmatized than physical health conditions. All physical health conditions in the RUSH-I except obesity were perceived as having relatively little stigma. Obesity, however, was perceived as more stigmatized than not only any other physical health condition but also any mental health condition. This was a striking and novel finding.

Among mental health conditions, paraphilias were perceived as the most stigmatized. This finding aligns with prior research demonstrating intense stigma towards even non-offending individuals with paraphilias, especially pedophilia (Lehmann et al., 2021). The stigmatization of paraphilias is especially concerning because it may dissuade sufferers from seeking treatment and, in turn, increase their likelihood of perpetration in the future. Consistent with prior studies (e.g., Barry et al., 2014; Corrigan et al., 2009; Crisp et al., 2000; Wood et al., 2014), other mental disorders that ranked high in stigma were substance use disorders, schizophrenia, and eating disorders. These diagnoses, as well as obesity, should be targets for stigma interventions.

Personality disorders, on the other hand, ranked unexpectedly low in stigma. Previous research has found that personality disorders, especially BPD and ASPD, are among the most stigmatized psychiatric conditions (Hazell et al., 2022). Unlike the RUSH-I, however, instruments used in prior research included symptom vignettes in addition to providing the name of each disorder. This distinction is especially important for personality disorders, as the public is largely unfamiliar with them (Furnham & Winceslaus, 2011). The names of personality disorders are also ambiguous, as in the case of BPD—or even potentially misleading, as in the case of ASPD; among the lay public, the term “antisocial” is often used to mean socially withdrawn. This misinterpretation could contribute to the especially low perceived stigma of ASPD in our sample, despite studies with symptom descriptions finding that it is highly stigmatized. Overall, personality disorders provide an example of how lack of knowledge could lead to lower awareness of stigma, especially if individuals are entirely unfamiliar with the symptoms of a disorder and the stereotypes and social attitudes associated with them.

This study was the first (to our knowledge) to compare stigma about perpetrating harmful behaviors to stigma about experiencing or being a survivor of those behaviors. As hypothesized, we found that perpetration was perceived as substantially more stigmatized than surviving such experiences. In fact, perpetration behaviors were perceived as more stigmatized than any other type of condition/behavior in the RUSH-I. While less stigmatized than perpetration, experiencing victimization was still associated with substantial stigma—more stigma, on average, than having a physical health or mixed condition. Being victimized as an adult was perceived as more stigmatized than being the victim of a similar act as a child, a discrepancy that may be explained by the fact that being victimized as an adult was also perceived as more controllable.

The limitations of the current study provide important context for the interpretation of these findings. Though we utilized a large, representative sample and performed sophisticated data cleaning to bolster data validity, there are limitations inherent in our cross-sectional, online survey-based methodology. The reliance on self-reporting may lead to biases like social desirability or inaccuracies due to misinterpretation of questions. Additionally, the cross-sectional nature of the study design precludes any causal inferences. The observed relationships between controllability and stigma, along with knowledge and stigma, at both the individual and group level could be influenced by omitted variables (e.g., perceived dangerousness or contagion worries). Reverse causality also cannot be ruled out; it is possible that stigma affects perceptions of controllability and reported knowledge. Furthermore, perceptions of controllability and knowledge may themselves be causally related. The nature of the relationships among these variables must be disentangled using experimental research. For instance, to determine whether differences in the perceived controllability of conditions are responsible for differences in stigma about them, researchers could examine whether interventions that close the controllability gap between these conditions also close the gap in stigma. Additional variables that may influence stigma should also be explored, especially factors that might explain the stigma gap between mental and physical health issues, which is not fully explained by knowledge and controllability. Future research should also investigate whether our findings about the relative stigma of health conditions/behaviors and their correlates replicate when key variables are measured differently. For instance, future studies could measure endorsements of stigma about various health conditions rather than perceptions of public stigma about them. They could also provide vignettes describing the symptoms of each condition in addition to or in place of providing condition names. Researchers might even explicitly compare how absolute and relative stigma about health issues vary when looking at perceptions versus endorsements and symptoms vignettes versus disorder names. Such work could be especially illuminating for stigma about personality disorders and other conditions with which the public is less familiar. Research should also reexamine the relationship between knowledge and stigma using objective assessments of participants’ understanding of each health issue.

Despite the present study’s limitations, it makes several important contributions to the stigma literature. While previous research has shown that knowledge and perceived controllability influence stigma, no previous work has established the degree to which these factors explain variations in stigma among specific health conditions. This study showed that these two factors strongly predict condition-specific stigma, collectively explaining over two-thirds of variation in perceived public stigma among 36 health problems and behaviors. A variety of conditions and behaviors that have not previously been directly compared were included, and thus the results generated new insights about their relative stigmatization. For instance, though the stigma of obesity has been documented in previous research (e.g., Hilbert et al., 2008; Weiner et al., 1988), this study presents novel evidence that it is perceived as more stigmatized than any mental health issue. Findings from this study have important implications for public health messaging and stigma-reduction campaigns. Interventions may be most impactful if they focus on conditions that are highly stigmatized and perceived as highly controllable, such as obesity, substance use disorders, and paraphilias. Messaging focused on reducing perceived personal blame while providing accurate information could help shift public attitudes and put these highly stigmatized issues on a more level playing field with other health conditions.

## Data Availability

No datasets were generated or analysed during the current study.
